# Effect of Tai Chi on the pain intensity or disability of patients with chronic low back pain: a systematic review and meta-analysis

**DOI:** 10.3389/fspor.2026.1676045

**Published:** 2026-02-12

**Authors:** Xiaohan Li, Kai Tang, Yuting Zhang, Lifeng Tang, Kun Wei, Min Tang

**Affiliations:** 1 Neurological Rehabilitation Department, Ningbo Rehabilitation Hospital, Ningbo, China; 2 Physical Education Department, Shenyang University of Chemical Technology, Shenyang, China; 3 Faculty of Marine, Ningbo University, Ningbo, China

**Keywords:** chronic pain, exercise therapy, low back pain, quality of life, Tai Chi

## Abstract

**Objectives:**

Chronic low back pain (CLBP) is a leading cause of disability worldwide and remains challenging to manage despite numerous treatment options. Tai Chi (TC), a traditional mind–body exercise, has been increasingly used as a non-pharmacological approach for CLBP, but previous trials and reviews have reported inconsistent results, particularly regarding disability outcomes and the influence of different TC parameters. This systematic review and meta-analysis aimed to synthesize current evidence on the effects of TC on pain intensity, disability, and other health-related outcomes in adults with CLBP, and to explore the potential impact of TC styles and training characteristics.

**Methods:**

Randomized controlled trials (RCTs) comparing TC with control interventions in adults with CLBP were searched in six English and Chinese databases from inception to May 2025. Primary outcomes were pain intensity and disability; secondary outcomes included physical function, general health, muscle function, and proprioception. Risk of bias was assessed using the Cochrane Risk of Bias tool, and the certainty of evidence was rated with the Grading of Recommendations Assessment, Development, and Evaluation (GRADE) approach. Where appropriate, meta-analyses and subgroup and sensitivity analyses were conducted. Publication bias was examined using funnel plots and Egger's regression test.

**Results:**

Fourteen RCTs (*n* = 960) met the inclusion criteria. Compared with control conditions, TC was associated with reduced pain intensity (SMD = −2.14; 95% CI: −2.84 to −1.44; *P* < 0.00001; GRADE: moderate) and improvements in disability measures (SMD = −1.45; 95% CI: −2.49 to −0.40; *P* = 0.007; GRADE: very low) and nine disability-related subdomains (pain intensity, personal care, lifting, walking, standing, sitting, sleeping, social life, and traveling). Benefits were also observed for physical function, muscle function, and quality of life, although evidence for proprioception was inconsistent. Subgroup analyses suggested that Chen-style TC and higher training frequency (>3 times/week) might be associated with larger pain reductions, but heterogeneity across studies remained substantial and the certainty of evidence ranged from moderate for pain intensity to very low for most disability outcomes.

**Conclusion:**

TC may be a safe and potentially effective adjunctive intervention for individuals with CLBP, with clinically relevant reductions in pain and improvements in physical function for some patients. However, substantial heterogeneity, small sample sizes in several trials, and low certainty of evidence for disability outcomes warrant cautious interpretation. Further high-quality, adequately powered RCTs with standardized TC protocols are needed to confirm these findings and to clarify the optimal style and training dose of TC for CLBP management.

**Systematic Review Registration:**

PROSPERO CRD420251072382.

## 1 Introduction

Chronic low back pain (CLBP) is defined as pain localized between the 12th rib and the inferior gluteal folds that persists for more than 3 months, with or without leg pain (sciatica) (1). It affects up to 23% of the adult population and is a leading cause of disability worldwide (2). Beyond its high prevalence, CLBP poses a persistent clinical challenge due to its multifactorial etiology, encompassing biomechanical (e.g., muscular weakness, poor spinal alignment), psychosocial (e.g., anxiety, catastrophizing), and neurophysiological (e.g., central sensitization) factors (3, 4). Conventional management strategies, including pharmacotherapy, physical therapy, and behavioral interventions, often yield only modest and short-term benefits, are associated with high recurrence rates, and contribute to rising healthcare burdens globally (4). This unmet clinical need has driven growing interest in complementary and alternative therapies (CATs) as potential adjunctive or standalone approaches for CLBP management.

Among CATs, Tai Chi (TC), a traditional Chinese mind-body practice that integrates slow, deliberate movements, rhythmic diaphragmatic breathing, and mindful focus, has emerged as a promising intervention for chronic pain conditions (5, 6). Unlike isolated physical exercises that primarily target biomechanical impairments, TC's holistic design addresses both the physical and psychosocial dimensions of CLBP. Its gentle movements enhance muscular strength, flexibility, and proprioception, which are key physical deficits in CLBP, whereas its meditative components mitigate stress, anxiety, and sleep disturbances, comorbidities that often exacerbate pain and disability in this population (6, 7). This dual mechanism aligns with contemporary biopsychosocial models of pain, making TC a theoretically robust candidate for CLBP management (8, 9). However, despite its conceptual appeal, the empirical evidence supporting TC's efficacy for CLBP remains fragmented.

In recent years, some randomized controlled trials (RCTs) have examined the efficacy of TC in alleviating pain and improving functional outcomes among individuals with CLBP (10, 11). However, inconsistencies in intervention protocols, study quality, outcome measures, and sample sizes have led to inconclusive results (12). Consequently, a number of reviews have evaluated TC or related mind–body exercises for CLBP. For example, Cui et al. published a protocol for a network meta-analysis of various non-pharmacological Chinese medicine therapies (including TC) for nonspecific CLBP, with the primary aim of comparing and ranking multiple interventions rather than evaluating TC in depth as a standalone therapy (13). Xia et al. compared the effects of four mind–body exercises (Qigong, yoga, TC, and guided imagery) on pain, physical function, and quality of life in adults with nonspecific CLBP (14). More recently, Sotiropoulos et al. synthesized RCTs of Qigong and TC for CLBP and primarily reported pooled effects on overall pain and disability outcomes (15). Although these reviews provide important evidence supporting mind–body interventions for CLBP, most were designed to evaluate a broad range of interventions or multiple mind–body modalities, with TC included as only one component, and they did not specifically focus on TC as a standalone intervention for CLBP. In addition, Chinese-language RCTs and theses remain underrepresented, and previous reviews have rarely examined in depth how specific intervention characteristics (e.g., TC style, session, duration, and frequency) may influence treatment effects.

Against this background, this systematic review and meta-analysis aims to address this gap by: (1) systematically evaluating the effects of TC on pain intensity and disability (primary outcomes) in adults with CLBP using data from RCTs published in English or Chinese and (2) exploring secondary outcomes (physical function, general health, muscle function, and proprioception) to provide a holistic understanding of TC's therapeutic mechanisms and potential. By synthesizing the available evidence and addressing the limitations of prior work, this study seeks to advance knowledge in CLBP management and inform evidence-based clinical decision-making.

## 2 Materials and methods

### 2.1 Protocol registration

This review was conducted and reported according to the Cochrane Collaboration Guideline and Preferred Reporting Items for Systematic Reviews and Meta-Analyses (PRISMA) recommendations (16). The study protocol was prospectively registered in the PROSPERO database (Registration number: CRD420251072382).

### 2.2 Search strategy

Following the PRISMA recommendations, a comprehensive search strategy was developed. Six electronic databases were systematically searched: Web of Science, MEDLINE, Cochrane Library, EMBASE, China National Knowledge Infrastructure (CNKI), and PubMed. The search period covered all available records up to May 2025, and studies published in either English or Chinese were considered. Search terms included combinations of “Tai Chi” and “Low back pain”. In addition, the reference lists of relevant articles and systematic reviews were manually screened to identify potentially eligible studies. Two independent reviewers (X.L. and K.T.) performed the literature search, and full search strategies are provided in Table 1.

**Table 1:** Search strategy on Web of science, MEDLINE, cochrane library, EMBASE, China national knowledge infrastructure (CNKI), and PubMed.

Search	Item
#1	(tai ji OR Tai-ji OR Tai Chi OR chi, tai OR Tai Ji Quan OR Ji Quan, Tai OR Quan Tai Ji OR taiji OR Taijiquan OR Tai chi OR Tai Chi Chuan OR shadowboxing)
#2	(Back Pain, Low OR Back Pains, Low OR Low Back Pains OR Pain, Low Back OR Pains, Low Back OR Lumbago OR Lower Back Pain OR Back Pain, Lower OR Back Pains, Lower OR Lower Back Pains OR Pain, Lower Back OR Pains, Lower Back OR Low Back Ache OR Ache, Low Back OR Aches, Low Back OR Back Ache, Low OR Back Aches, Low OR Low Back Aches OR Low Backache OR Backache, Low OR Backaches, Low OR Low Backaches OR Postural Low Back Pain OR Recurrent Low Back Pain OR Mechanical Low Back Pain OR Chronic Non-Specific Low Back Pain)
#3	(Pain OR Pain Intensity OR Disability OR VAS OR Visual Analogue Scale OR NRS OR Numerical Rating Scale OR RMDQ OR Roland-Morris Disability Questionnaire OR ODI OR Oswestry Disability Index OR Function OR Functional Limitation OR Functional Status OR Mobility OR Muscle Strength OR Balance OR Proprioception OR Gait)
#4	(Randomized Controlled Trial OR Randomized OR Randomised OR RCT)
#5	1 AND 2 AND 3 AND 4

### 2.3 Eligibility criteria

#### 2.3.1 Inclusion criteria

Studies were selected based on the PICOS framework as follows:

(a) Population (P): Adults diagnosed with CLBP, defined as musculoskeletal pain between the 12th rib and the gluteal fold lasting more than 3 months, with or without radiating leg pain (sciatica) (1).
(b) Intervention (I): The experimental group (EG) received TC exercise without any additional treatments (e.g., pharmacotherapy, manual therapy, or cognitive behavioral therapy). In all included trials, TC interventions were delivered in group classes led by experienced TC instructors and, in some studies, were supplemented by home practice. The most commonly used forms were simplified 24-style TC (derived from Yang-style TC), Chen-style TC, Sun-style TC, and a “flashback” method focusing on lumbar and trunk movements. Simplified 24-style TC emphasizes slow, continuous, and symmetrical movements with upright postures and coordinated diaphragmatic breathing (17). Chen-style TC is characterized by lower stances, spiral and rotational movements of the trunk, and alternating fast–slow tempos, which may place greater demands on lower-limb strength and neuromuscular control (18). Sun-style TC generally involves higher stances, smooth step-forward and step-backward patterns, and coordinated arm movements with relaxed breathing, which may be more suitable for older adults (19). The “flashback” method combines semi-squat postures and repeated lumbar flexion–extension and rotation patterns designed to enhance spinal stability (20). Across studies, each TC session typically included a brief warm-up, practice of the TC routine, and a cool-down phase with relaxation and breathing exercises.
(c) Comparison (C): The control group (CG) received no intervention, or received conventional training, or received an alternative intervention such as health education.
(d) Outcomes (O): The primary outcomes included pain intensity [e.g., measured using Visual Analogue Scale (VAS) or Numerical Rating Scale (NRS)] and disability [e.g., measured using the Roland Morris Disability Questionnaire (RMDQ) or the Oswestry Disability Index (ODI)]. Secondary outcomes included physical function, general health, muscle function, and proprioception.
(e) Study Design (S): Only RCTs were included.

#### 2.3.2 Exclusion criteria

(a) Studies that reported duplicate data from the same research cohort or that were secondary analyses of primary studies already included.
(b) Studies that did not provide sufficient quantitative data for at least one primary outcome (pain intensity or disability) at post-intervention, and for which the required information could not be obtained from the authors.
(c) Studies published only as conference abstracts, research protocols, books, or other non–peer-reviewed reports.

### 2.4 Study selection

Two independent reviewers (X.L. and K.T.) screened all retrieved articles based on titles and abstracts. For studies with unclear eligibility from the abstract, full-text screening was conducted. Disagreements were resolved through discussion, and unresolved discrepancies were adjudicated by a third reviewer (M.T.).

### 2.5 Data extraction

Two reviewers (X.L. and Y.Z.) independently extracted data using a pre-defined data extraction form. Extracted information included: publication information (author, year, and country), subject characteristics (sample size, age, and disease course), EG characteristics (TC style, duration, session, frequency), CG characteristics, and outcomes. In cases where multiple time points were reported, only data immediately post-intervention were used for meta-analysis. Disagreements were resolved by consensus.

### 2.6 Risk of bias and GRADE

Risk of bias was assessed using the Cochrane Risk of Bias Tool in RevMan 5.4.1 (The Cochrane Collaboration, Oxford, UK) (21). Seven domains were evaluated: random sequence generation, allocation concealment, blinding of participants and personnel, blinding of outcome assessment, incomplete outcome data, selective reporting, and other biases. Each aspect was rated by the researchers as high risk (−), low risk (+), or uncertain risk (?). In cases of disagreement on the ratings, a consultation process was implemented to reach a consensus.

The certainty of evidence for each outcome was assessed using the GRADE (Grading of Recommendations Assessment, Development, and Evaluation) approach  (22). Two reviewers (L.T. and K.W.) independently evaluated five factors that may downgrade the certainty of evidence: risk of bias or limitations in study design and implementation; unexplained heterogeneity or inconsistency of results; indirectness of the evidence; imprecision of results; and a high probability of publication bias. The certainty level was classified as high, moderate, low, or very low. Discrepancies were resolved through discussion.

### 2.7 Data synthesis and analysis

All statistical analyses were conducted using RevMan (version 5.4.1; The Cochrane Collaboration, Oxford, UK). For continuous outcomes, standardized mean differences (SMDs) with 95% confidence intervals (CIs) were calculated. When studies used different pain intensity scales (e.g., VAS and NRS), SMDs were used to allow pooling of results. Statistical heterogeneity was assessed using the I² statistic, with values of <25%, 26%–74%, and ≥75% representing low, moderate, and high heterogeneity, respectively (23). In line with these thresholds, a fixed-effect model was applied when heterogeneity was low (*I*^2^ < 25%), whereas a random-effects model was used when heterogeneity was ≥25% (23). For pain intensity, in addition to the overall meta-analysis, subgroup analyses were prespecified and performed according to TC style, duration, session, frequency (Table 2), and type of pain rating scale (VAS vs. NRS) to explore potential sources of heterogeneity. For disability outcomes (RMDQ and ODI, including its subdomains), subgroup analyses were not conducted because of the limited number of available studies.

**Table 2:** Subgroup classification criteria.

Topics	Categories
TC style	24-Style; Chen-Style; Sun-Style; Other/NR
Duration (weeks)	≤10 weeks; >10 weeks
Session (min)	≤45 min; >45 min; Other/NR
Frequency (times/week)	≤3 times/week; >3 times/week

NR, Not Report; TC, Tai Chi.

Potential publication bias was evaluated using funnel plots and Egger's regression test when at least 10 studies were available for a given outcome (24). When publication bias was suspected, the “trim-and-fill” method was applied to assess the robustness of the results (25). For the primary outcomes, leave-one-out sensitivity analyses were performed to evaluate the influence of individual studies on the overall results.

## 3 Results

### 3.1 Search results and study quality

**Figure 1:**
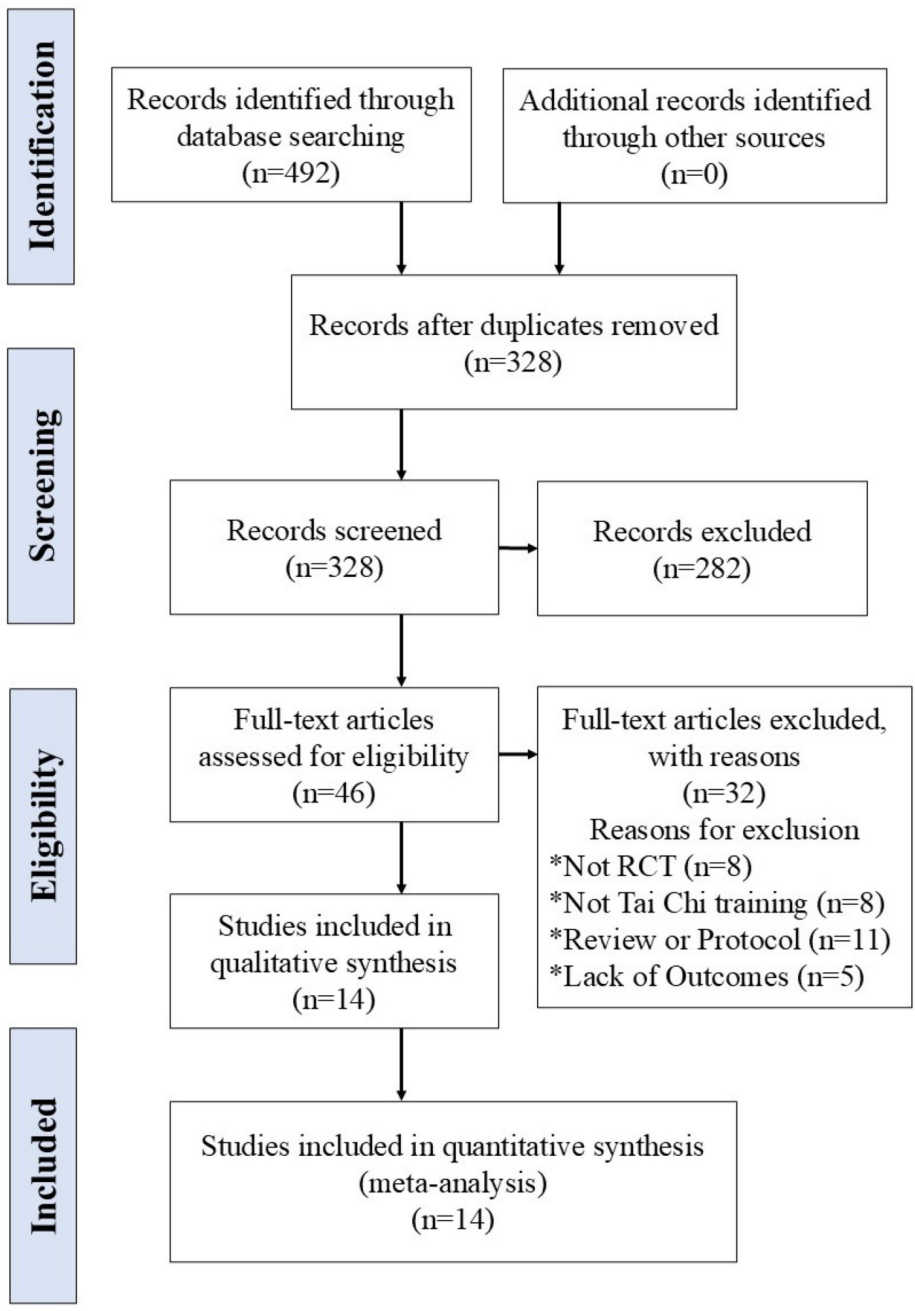
Flowchart of literature selection.

The detailed flow diagram of the study selection process is presented in Figure 1. A total of 492 potentially relevant records were identified through systematic searches of both English and Chinese electronic databases. After removing 164 duplicates, 328 records remained for eligibility screening. Of these, 282 articles were excluded due to irrelevance based on their titles and abstracts. Forty-six full-text articles were then assessed for eligibility. Subsequently, 32 studies were excluded for the following reasons: Not RCTs (*n* = 8), TC was not the main intervention (*n* = 8), Article type was review or protocol (*n* = 11), No usable data reported for analysis (*n* = 5). As a result, 14 RCTs (10, 11, 17–20, 26–33) were included in the meta-analysis. Among them, 6 studies (10, 11, 19, 26, 28, 32) were published in English, and 8 (17, 18, 20, 27, 29–31, 33) in Chinese.

### 3.2 Study characteristics

Table 3 provides an overview of the characteristics of the included studies. All 14 included studies (10, 11, 17–20, 26–33) were RCTs, targeting either CLBP or non-specific CLBP. Collectively, these studies involved 960 participants, with 532 in the EG and 428 in the CG. In terms of TC style: four studies (10, 17, 27, 31) employed the 24-Style TC, seven studies (11, 18, 26, 28, 29, 32, 33) used the Chen-Style, one study (19) adopted the Sun-Style, the remaining studies (20, 30) utilized other unspecified styles. The training frequency ranged from 1 to 6 times per week, with individual session durations varying between 30 and 60 min. The total intervention duration across studies ranged from 4 weeks to 24 weeks.

**Table 3:** Characteristics of the included studies.

Study (arthor, year)	Country (language)	Sample (EG/CG, n)	Age (EG/CG, years)	Disease course (EG/CG)	TC style	Duration (weeks)	Session (min)	Frequency (times/week)	CG intervention	Outcomes
Hall et al. (19)	Australia (English)	80/80	43.4 ± 13.5/44.3 ± 13.0	>3 months	Sun-Style	10	40	1–8 weeks: 2; 9–10 weeks: 1	Usual health care	②③⑤
He (18)	China (Chinese)	16/13	58.56 ± 4.23/59.38 ± 4.78	>3 months	Chen-Style	12	60	3	Normal daily activities	④
Li (17)	China (Chinese)	13/12	21.08 ± 1.26/20.75 ± 1.14	6.54 ± 1.21/5.74 ± 1.16 (months)	24-Style	8	45	3	Normal daily activities	①③⑤⑦
Liu et al. (26)	China (English)	15/13	58.13 ± 5.38/60.67 ± 2.58	>3 months	Chen-Style	12	60	3	Normal daily activities	①⑧
Lu et al. (27)	China (Chinese)	54/54	63.37 ± 6.59/67.97 ± 6.00	4.97 ± 4.73/4.74 ± 3.84 (years)	24-Style	12	40	5	Drug treatment	①⑥
Muharram et al. (28)	China (English)	82/82	43.6 ± 3.32/43.3 ± 3.67	3.0 ± 0.87/3.1 ± 0.77 (years)	Chen-Style	12	60	6	Health education	①⑥
Tian (29)	China (Chinese)	15/13	58.13 ± 5.38/60.67 ± 2.58	NR	Chen-Style	12	60	3	Normal daily activities	①⑦⑧
Tong et al. (30)	China (Chinese)	32/32	32.60 ± 6.46/32.66 ± 6.53	NR	NR	4	NR	2	Rest on the hard board bed	①④
Tong (20)	China (Chinese)	35/36	42.3 ± 4.26/41.6 ± 4.09	>3 months	“flashback”	12	30	3	Strength exercise	①⑦
Wang (31)	China (Chinese)	10/10	65.10 ± 3.57/62.30 ± 3.65	13.10 ± 7.92/11.60 ± 5.78 (years)	24-Style	12	60	3	Physical therapy	②③
Weifen et al. (32)	China (English)	141/47	37.5 ± 5.2/38.1 ± 5.2	2.10 ± 0.74/2.2 ± 0.81 (years)	Chen-Style	24	45	5	No any regular sports activities	①⑥
Yan et al. (10)	China (English)	10/10	68.00 ± 1.15/70.00 ± 1.26	>3 months	24-Style	6	60	3	Normal daily activities	①⑤
Zhao (33)	China (Chinese)	14/13	58.92 ± 3.65/57.35 ± 2.97	NR	Chen-Style	12	60	3	Normal daily activities	④
Zou et al. (11)	China (English)	15/13	58.13 ± 5.38/60.67 ± 2.58	NR	Chen-Style	12	60	3	Normal daily activities	①⑦

CG, control group; EG, experimental group; NR, not report; NRS, numeric rating scale; ODI, oswestry disability index; RMDQ, roland morris disability questionnaire; TC, Tai Chi; VAS, visual analogue scale. Outcomes: ① Pain Intensity (VAS), ② Pain Intensity (NRS), ③ Disability (RMDQ), ④ Disability (ODI), ⑤ Physical Function, ⑥ General Health, ⑦ Muscle Function, ⑧ Proprioception.

### 3.3 Risk of bias assessment

**Figure 2:**
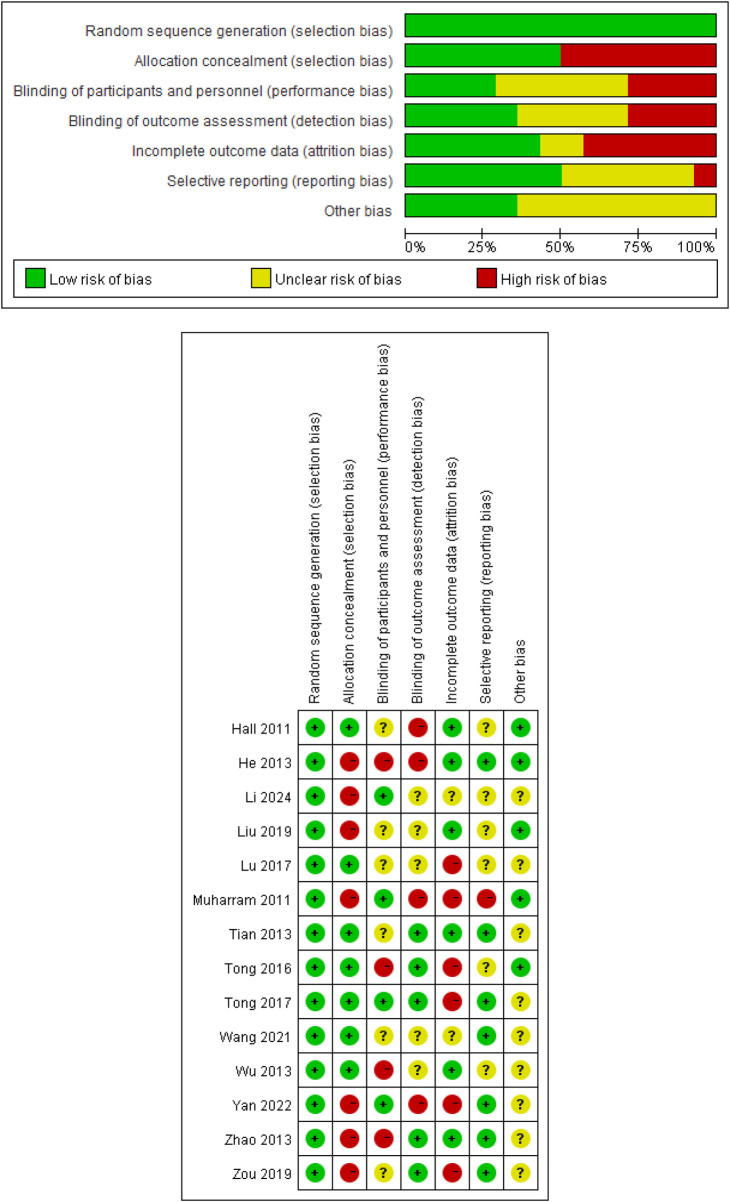
Evaluation of the quality of the included literature.

The risk-of-bias assessments for each study and domain are summarized in Figure 2. All trials (10, 11, 17–20, 26–33) were judged to be at low risk of bias for random sequence generation, whereas allocation concealment was rated as high risk in approximately half of the trials (10, 11, 17, 18, 26, 28, 33). Because TC is a behavioral intervention, blinding of participants and personnel was generally not feasible, leading to a high risk of performance bias in several studies (18, 30, 32, 33). Blinding of outcome assessment was more frequently reported and was usually rated as low or unclear risk (11, 17, 20, 26, 27, 29–33). Incomplete outcome data were judged to be at high risk of bias in some trials (10, 11, 20, 27, 28, 30), and selective reporting was generally well managed, with most studies rated as having low or unclear risk in this domain (10, 11, 17–20, 26, 27, 29–33). Overall, most trials had at least one domain with high or unclear risk of bias.

### 3.4 Primary outcomes

#### 3.4.1 Effect of Tai Chi in pain intensity

**Figure 3:**
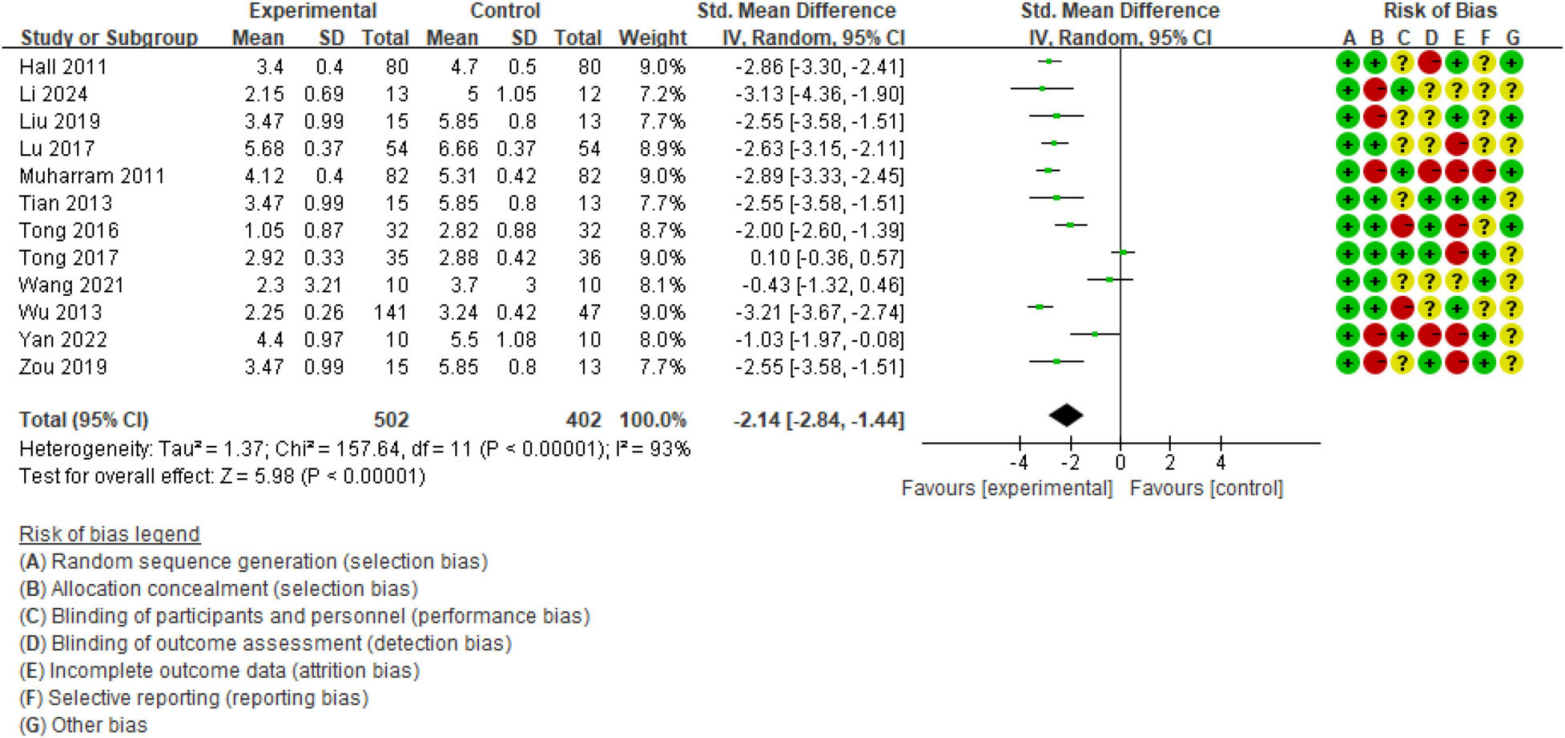
Forest plot of effect of Tai Chi for pain intensity.

A total of 12 RCTs (10, 11, 17, 19, 20, 26–32) involving 904 participants were included in the meta-analysis of pain intensity outcomes. The assessment tools for pain intensity varied among studies: 10 studies (10, 11, 17, 20, 26–30, 32) used a 0–10 mm VAS, and 2 studies (19, 31) used a 0–10 NRS. As shown in Figure 3, TC was associated with a reduction in pain intensity compared with the CG (SMD = −2.14, 95% CI −2.84 to −1.44; *P* < 0.00001), although substantial heterogeneity was present (*I*^2^ = 93%; GRADE: moderate, Supplementary Table S1).

To explore potential sources of heterogeneity, exploratory subgroup analyses for pain intensity were conducted according to TC style, duration, session, frequency, and type of pain rating scale (Table 4; Supplementary Figures S1–S5). For TC style, the test for subgroup differences was significant (*P* < 0.00001). Trials using Chen-style TC showed the largest effect with minimal heterogeneity (SMD = −2.93, 95% CI: −3.21 to −2.65; *I*^2^ = 0%), followed by simplified 24-style TC (SMD = −2.00, 95% CI: −2.39 to −1.62; *I*^2^ = 88%) and other/not report (NR) styles (SMD = −1.57, 95% CI: −1.85 to −1.29; *I*^2^ = 98%). Subgroup analyses by duration (≤10 vs. >10 weeks) and session (≤45 vs. >45 min) did not show significant subgroup differences (*P* = 0.80 and *P* = 0.91, respectively), and heterogeneity remained substantial within all categories (*I*^2^ = 80%–97%). In contrast, frequency appeared to partly explain heterogeneity: programs delivered more than three times per week showed a larger effect and lower heterogeneity (SMD = −2.92, 95% CI: −3.24 to −2.61; *I*^2^ = 25%) than programs with three or fewer sessions per week (SMD = −1.86, 95% CI: −2.77 to −0.95; *I*^2^ = 93%; P for subgroup difference = 0.03).

**Table 4:** Subgroup analysis results of pain intensity.

Subgroup		Study quantity (n)	Sample (n)		SMD (95% CI)	Heterogeneity effect		Overall effect		Subgroup difference
Topics	Categories		EG	CG		*I* ^2^	*P*	Z	*P*	
TC Style	24-Style	4	87	86	−2.00 (−2.39, −1.62)	88%	<0.00001	10.19	<0.00001	<0.00001
	Chen Style	5	268	168	−2.93 (−3.21, −2.65)	0%	0.56	20.37	<0.00001	
	Other/NR	3	147	148	−1.57 (−1.85, −1.29)	98%	<0.00001	10.84	<0.00001	
Duration (weeks)	≤10 weeks	4	135	134	−2.25 (−3.07, −1.43)	80%	0.002	5.35	<0.00001	0.80
	>10 weeks	8	367	268	−2.08 (−3.08, −1.09)	95%	<0.00001	4.11	<0.00001	
Session (min)	≤45 min	5	323	229	−2.32 (−3.66, −0.98)	97%	<0.00001	3.40	0.0007	0.91
	>45 min	6	147	141	−2.01 (−2.87, −1.14)	84%	<0.00001	4.56	<0.00001	
	Other/NR	1	32	32	−2.00 (−2.60, −1.39)	/	/	6.46	<0.00001	
Frequency (times/week)	≤3 times/week	9	225	219	−1.86 (−2.77, −0.95)	93%	<0.00001	4.01	<0.00001	0.03
	>3 times/week	3	277	183	−2.92 (−3.24, −2.61)	25%	0.26	18.11	<0.00001	
Rating Scale	VAS	10	412	312	−2.23 (−3.03, −1.43)	93%	<0.00001	5.45	<0.00001	0.67
	NRS	2	90	90	−1.69 (−4.04, 0.65)	96%	<0.00001	1.41	0.16	

CG, control group; CI, confidence interval; EG, experimental group; NR, not report; NRS, numeric rating scale; SMD, standardized mean difference; TC, Tai Chi; VAS, visual analogue scale.

Because pain intensity was assessed using two different scales, we also conducted a subgroup analysis by pain rating scale. The VAS subgroup (10 studies) showed a pooled SMD of −2.23 (95% CI: −3.03 to −1.43; *I*^2^ = 93%), whereas the NRS subgroup (2 studies) showed an SMD of −1.69 (95% CI: −4.04 to 0.65; *I*^2^ = 96%). The test for subgroup differences between VAS and NRS was not significant (*P* = 0.67), indicating that the choice of pain scale alone does not account for the substantial heterogeneity observed.

#### 3.4.2 Effect of Tai Chi in disability

**Figure 4:**
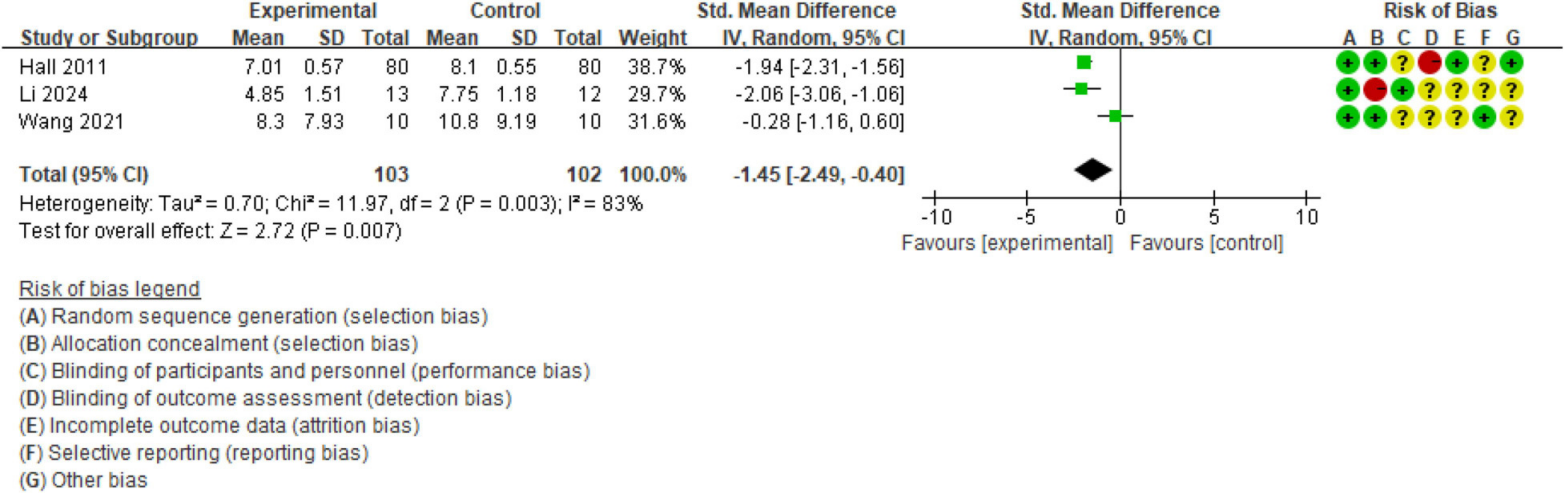
Forest plot of effect of Tai Chi for RMDQ.

Disability was assessed using the RMDQ and ODI. Three studies (17, 19, 31) (*n* = 205) reported RMDQ scores. As shown in Figure 4, the pooled analysis suggested that TC was associated with a reduction in disability compared with the CG (SMD = −1.45, 95% CI: −2.49 to −0.40; *P* = 0.007), although heterogeneity was substantial (*I*^2^ = 83%) and the certainty of evidence was rated as very low (Supplementary Table S1).

**Figure 5:**
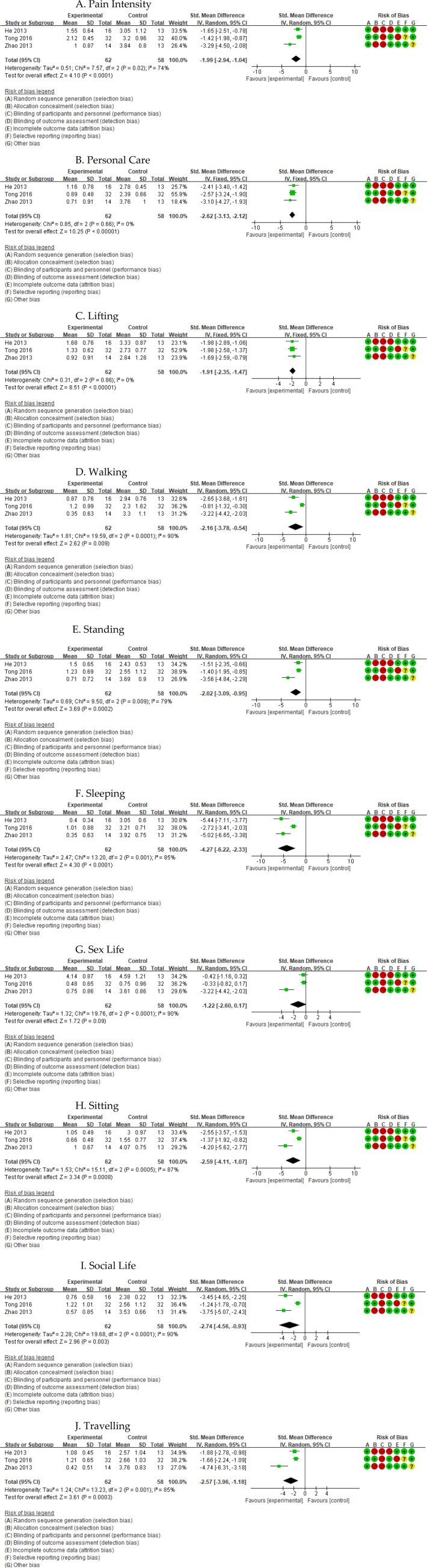
Forest plot of effect of Tai Chi for ODI. **(A)** Pain Intensity. **(B)** Personal Care. **(C)** Lifting. **(D)** Walking. **(E)** Standing. **(F)** Sleeping. **(G)** Sex Life. **(H)** Sitting. **(I)** ocial Life. **(J)** Travelling.

Three studies (18, 30, 33) reported ODI scores and allowed analysis of ten subdomains: pain intensity, personal care, lifting, walking, standing, sleeping, sitting, social life, traveling, and sex life (Figure 5). TC was associated with greater improvements than the CG in nine subdomains—pain intensity (SMD = −1.99, 95% CI: −2.94 to −1.04; *I*^2^ = 74%), personal care (SMD = −2.62, 95% CI: −3.13 to −2.12; *I*^2^ = 0%), lifting (SMD = −1.91, 95% CI: −2.35 to −1.47; *I*^2^ = 0%), walking (SMD = −2.16, 95% CI: −3.78 to −0.54; *I*^2^ = 90%), standing (SMD = −2.02, 95% CI: −3.09 to −0.95; *I*^2^ = 79%), sleeping (SMD = −4.27, 95% CI: −6.22 to −2.33; *I*^2^ = 85%), sitting (SMD = −2.59, 95% CI: −4.11 to −1.07; *I*^2^ = 87%), social life (SMD = −2.74, 95% CI: −4.56 to −0.93; *I*^2^ = 90%), and traveling (SMD = −2.57, 95% CI: −3.96 to −1.18; *I*^2^ = 85%)—whereas no statistically significant between-group difference was observed for sex life (SMD = −1.22, 95% CI: −2.60 to 0.17; *I*^2^ = 90%; *P* = 0.09). With the exception of personal care and lifting, heterogeneity for ODI subdomains was high, and the certainty of evidence was rated as very low for most domains (low for personal care and lifting) (Supplementary Table S1).

Given the small number of studies contributing to RMDQ and ODI outcomes, we did not perform subgroup analyses for disability, and the pooled estimates should be interpreted cautiously in view of the substantial heterogeneity and low certainty of evidence.

### 3.5 Secondary outcomes

A total of 12 distinct secondary outcomes were reported across four domains: physical function (6 outcomes), general health (2 outcomes), muscle function (3 outcomes), and proprioception (1 outcome) (Table 5). Because outcome measures and assessment protocols varied widely and most outcomes were reported by only one or two trials, quantitative pooling was not appropriate; these findings are therefore summarized narratively.

**Table 5:** Summary of outcomes not included in the meta-analysis.

Outcome variable	Study	Measurement method/instrument	Main results
Physical function			
Disability	Hall et al. (19)	PDI (0–70), QBPDS (20–100)	TC significantly reduced pain-related disability
Functional Status	Hall et al. (19)	PSFS (0–10)	Slight reduction in functional score
Perceived Recovery	Hall et al. (19)	GPE scales (−5 to +5)	Slightly greater perceived recovery
Flexibility	Li (17)	Goniometric lumbar mobility test (Yun Xiaoping's method)	TC significantly improved lumbar flexion, extension, lateral flexion (left/right), and rotation (left/right)
Spatiotemporal Gait Features	Yan et al. (10)	Gait analysis (step length, step width, gait speed)	Significant improvements in gait speed and step length
Dynamic Balancing Capacity	Yan et al. (10)	Single-leg balance, center of pressure shift test	Significant improvements in balance across multiple directions
General health			
Basic Physiology	Muharram et al. (28)	BMI, SBP, DBP, HR, Sleep	No significant effects
	Weifen et al. (32)		
Quality of life	Lu et al. (27)	SF-36	TC significantly improved both physical and mental health domains
	Muharram et al. (28)		
Muscle function			
Lower-limb neuromuscular function	Zou et al. (11)	Biodex dynamometer (peak torque and endurance at 60°/s, 180°/s)	Significant improvements in left knee and right ankle endurance
Spinal stability	Tong (20)	IsoMed2000 (30°/s, 60°/s trunk flexion/extension)	Trunk flexion-extension peak torque ratio significantly reduced
Muscle endurance	Li (17)	Static hold tests (supine leg raise, prone trunk extension)	TC significantly improved abdominal and back endurance
	Tian (29)	Biodex System 3 at 60°/s, 180°/s (5 reps per speed)	Improved peak torque (extensors/flexors) and endurance at 60°/s; minor endurance gain at 180°/s
Proprioception			
Liu et al. (26)		Biodex dynamometer (active JPS, angle error)	No significant improvement
Tian (29)		Biodex System 3 (JPS at 45°, 10°, under sensory isolation)	TC group showed improved proprioceptive accuracy

BMI, body mass index; DBP, diastolic blood pressure; GPE, global perceived effect; HR, heart rate; JPS, joint position sense; QBPDS, quebec back pain disability scale; PDI, pain disability index; PSFS, patient-specific functional scale; SBP, systolic blood pressure; TC, Tai Chi.

#### 3.5.1 Physical function

Six outcomes related to physical function were assessed, including disability, functional status, perceived recovery, flexibility, spatiotemporal gait features, and dynamic balancing capacity. One trial reported that TC clearly reduced pain-related disability and slightly improved functional status and perceived recovery (19). Another found significant gains in lumbar flexion, extension, lateral flexion, and rotation (17). In addition, TC improved gait speed, step length, and single-leg balance with centre-of-pressure control in one study (17).

#### 3.5.2 General health

Two studies (28, 32) evaluated basic physiological indices [e.g., body mass index (BMI), blood pressure, heart rate (HR), sleep] and found no consistent or clinically important effects of TC. Health-related quality of life was assessed in two trials (27, 28) using a generic questionnaire; in one of them, TC led to improvements in both physical and mental health domain.

#### 3.5.3 Muscle function

Three outcomes reflected muscle function: lower-limb neuromuscular function, spinal stability, and muscle endurance. Isokinetic dynamometry studies (11, 20) reported that TC increased peak torque and endurance at the knee and ankle and improved trunk flexion–extension torque ratios, indicating better spinal stability. Static endurance tests (17, 29) showed enhanced abdominal and back muscle endurance after TC training.

#### 3.5.4 Proprioception

Two studies (26, 29) examined proprioception using joint position sense (JPS) tests with isokinetic dynamometers. One trial (26) found no significant between-group differences in active joint position reproduction, whereas the other reported (29) better proprioceptive accuracy in the TC group under sensory-isolation conditions.

### 3.6 Publication bias and sensitivity analysis

**Figure 6:**
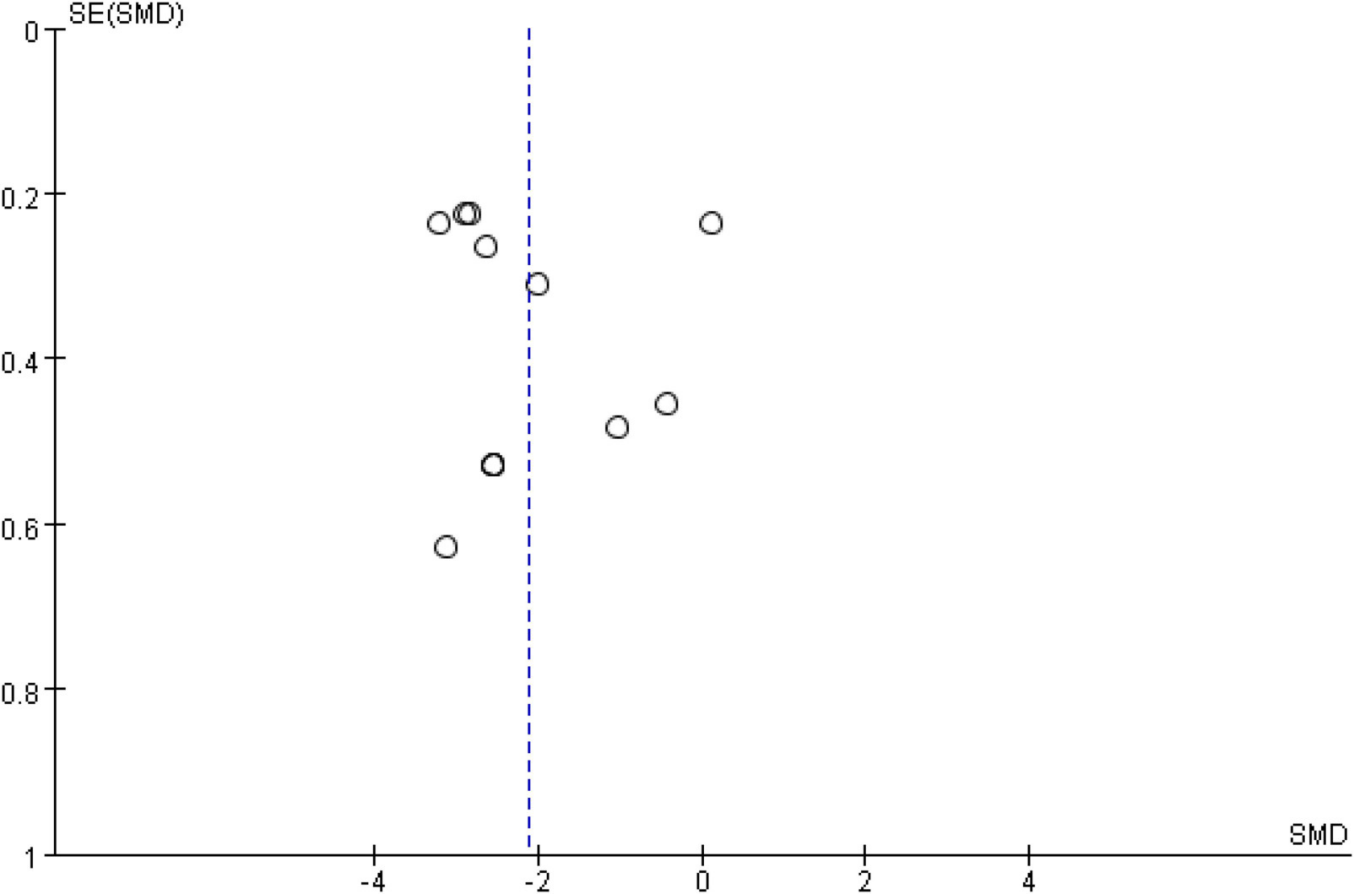
Funnel plot of pain intensity.

Publication bias for pain intensity was assessed using a funnel plot and Egger's regression test based on the 12 trials (10, 11, 17, 19, 20, 26–32) that reported VAS or NRS scores and contributed to the primary pain meta-analysis (Figure 6). Although the funnel plot showed some asymmetry, Egger's test did not indicate statistically significant small-study effects (*t* = 0.24, *P* = 0.818). The remaining two of the 14 included RCTs (18, 33) did not provide sufficient quantitative data for pain intensity and were therefore not included in this analysis.

Leave-one-out sensitivity analyses for pain intensity are presented in Supplementary Table S2. When each of the 12 trials (10, 11, 17, 19, 20, 26–32) was removed in turn, the pooled effect remained statistically significant and consistently favored TC, with SMDs ranging from −2.38 to −2.03 (all *P* < 0.00001), and heterogeneity remained high (*I*^2^ = 79%–94%). These results suggest that the overall pain-relieving effect was not driven by any single study, although considerable between-study heterogeneity persists. Sensitivity analyses were not performed for RMDQ or ODI outcomes because too few studies (*n* = 3) were available to meaningfully assess the robustness of the pooled estimates.

## 4 Discussion

This review synthesized data from 14 RCTs involving 960 participants to examine the effects of TC on pain intensity, disability, and related outcomes in adults with CLBP. Overall, TC was associated with reductions in pain intensity and improvements in back-related disability compared with control conditions, and additional benefits were observed for physical function, muscle performance, and quality of life. However, these findings should be interpreted cautiously in view of the substantial heterogeneity between studies, the small sample sizes of several trials, and the low or very low certainty ratings for most disability outcomes on the GRADE framework.

### 4.1 Effect of Tai Chi in pain intensity

The meta-analysis indicated that TC was associated with a clinically relevant reduction in pain intensity compared with various control interventions. The magnitude of the pooled effect was larger than commonly cited minimal clinically important difference (MCID) values for chronic pain scales (34, 35), suggesting that many patients may experience meaningful pain relief. These results are broadly consistent with previous reviews of TC and other mind–body exercises for CLBP (12, 36).

Potential mechanisms underlying the analgesic effects of TC include a combination of peripheral and central processes (37, 38). Slow, coordinated movements and postural control may improve local circulation, spinal stability, and neuromuscular function, while diaphragmatic breathing and mindful attention may modulate pain perception through reduced stress, improved emotional regulation, and engagement of descending inhibitory pathways (37, 38).

Subgroup analyses suggested that Chen-style TC and programs delivered more than three times per week tended to show larger pain reductions than other styles and lower-frequency programs. This may be due to the more complex and dynamic motor patterns involved in Chen-style TC, which may stimulate greater neuromuscular activation, proprioceptive input, and cortical engagement (35, 39). However, these subgroup findings were based on relatively few studies and small subgroup sample sizes, and substantial residual heterogeneity remained even within subgroups. In addition, pain was measured using different scales (VAS and NRS), and although our analyses did not detect meaningful differences between these scales, this variation may have contributed to between-study variability. Taken together, the available data suggest that TC may reduce pain intensity in CLBP, but the precise magnitude of benefit and the extent to which it depends on style or training dose remain uncertain and should be considered hypothesis-generating rather than prescriptive.

### 4.2 Effect of Tai Chi in disability

The RMDQ, a widely used tool for assessing functional limitations in CLBP (40), was used in several included studies (17, 19, 31). Our meta-analysis suggested that TC was associated with reductions in RMDQ scores, indicating potential improvements in daily functioning (e.g., walking, bending, dressing, and prolonged standing). Importantly, these improvements exceeded the MCID thresholds reported in prior literature (34), reinforcing the clinical relevance of the findings. However, these findings were based on limited sample sizes, accompanied by substantial heterogeneity, and the certainty of evidence for this outcome was rated as very low; therefore, the apparent functional benefits should be interpreted with caution.

Three studies (18, 30, 33) reported disability outcomes using the ODI and its subdomains. Pooled analyses showed statistically significant improvements in nine of ten subdomains—pain intensity, personal care, lifting, walking, standing, sitting, sleeping, social life, and traveling—whereas no significant change was observed for sex life. This pattern suggests that TC may have a broad impact on several physical and social aspects of back-related disability, while domains such as sexual functioning, which are strongly influenced by psychological, relational, and sociocultural factors, may be less responsive to exercise-based interventions alone (36, 41). Nonetheless, the ODI findings are again derived from only three trials with high between-study heterogeneity, and GRADE ratings were low for some subdomains and very low for most others. Because of the small number of available studies, we were unable to perform meaningful subgroup analyses for disability, and the pooled estimates should be regarded as preliminary. Further large, high-quality RCTs with standardized disability measures and detailed reporting of ODI subdomains are needed to confirm and better characterize the effects of TC on functional outcomes in CLBP.

### 4.3 Effect of Tai Chi in other functional and health-related outcomes

Beyond pain intensity and disability, TC may confer additional benefits across secondary domains, although the evidence base remains limited and largely derived from single trials or small samples. Individual studies (10, 17) reported improvements in lumbar mobility, gait parameters, and dynamic balance, which may be consistent with TC's emphasis on controlled weight shifting, trunk coordination, and mindful movement. Similarly, several trials (11, 17, 20, 29) suggested favorable changes in muscle-related outcomes, including lower-limb performance, trunk muscle balance, and core endurance, implying that TC may contribute to enhanced spinal support and neuromuscular control in people with CLBP.

In contrast, evidence for proprioceptive and general health effects was inconsistent. JPS outcomes varied across studies (26, 29), and basic physiological indices such as BMI, blood pressure, HR, and sleep duration did not show consistent improvements (28, 32). Quality of life appeared more responsive, with some evidence of gains in both physical and mental health domains (27, 28). However, given the heterogeneity of measurement methods and the very limited number of studies per outcome, these findings should be interpreted as exploratory and hypothesis-generating rather than definitive evidence of broad health benefits. Future well-powered RCTs with standardized outcome sets are needed to clarify the clinical significance of these secondary effects.

### 4.4 Clinical implications and limitations

From a clinical perspective, TC may be considered a promising adjunctive, non-pharmacological option within multimodal conservative management of CLBP. It is relatively low cost, requires minimal equipment, and can be delivered in community or home settings, which may facilitate adherence. Its combined focus on gentle physical activity, postural control, breathing, and mindful awareness is aligned with contemporary biopsychosocial models of chronic pain, potentially addressing both somatic and central contributors to CLBP. Nevertheless, given the limitations of the current evidence, TC should be integrated as part of an individualized treatment plan rather than viewed as a standalone cure, and specific recommendations regarding style or frequency cannot yet be made with confidence.

Nonetheless, several limitations warrant attention. First, most included studies were conducted in China, which may increase the risk of regional publication bias and limit the generalizability of the findings to other settings. Second, heterogeneity was substantial for pain intensity and disability outcomes and was not fully explained by subgroup analyses. Differences in TC protocols, comparator interventions, outcome measures, assessment timing, and participant characteristics likely contributed to this inconsistency. Third, many of the included RCTs had small sample sizes and were of moderate-to-very low methodological quality, which limits the strength and certainty of the evidence. Finally, although study selection was performed independently by two reviewers and disagreements were resolved through discussion, we did not formally quantify inter-rater agreement (e.g., using kappa statistics), which may introduce a small risk of selection bias. Future research should prioritize large, high-quality trials with standardized and well-described TC protocols, appropriate active comparators, and consistent outcome reporting to better establish the efficacy and sustainability of TC for CLBP.

## 5 Conclusion

This review suggests that TC may be a safe and potentially beneficial adjunctive intervention for adults with CLBP. Across the included trials, TC was associated with reductions in pain intensity and improvements in back-related disability, physical function, muscle function, and quality of life. Exploratory analyses indicated that Chen-style TC and programs delivered more than three times per week might be associated with larger pain reductions; however, these observations are based on a limited number of studies and substantial residual heterogeneity, and should therefore be interpreted as hypothesis-generating rather than as firm clinical recommendations.

Given the substantial heterogeneity across trials, the limited number of studies contributing to disability and most secondary outcomes, and the generally low to very low GRADE ratings for these findings, our results should be interpreted cautiously and should not be considered definitive evidence of efficacy. Future large, multicenter RCTs with standardized and well-described TC protocols, and appropriate active comparators are needed to determine the optimal style and training dose, clarify potential mechanisms, and strengthen the evidence base for clinical decision-making in CLBP.

## Data Availability

The raw data supporting the conclusions of this article will be made available by the authors, without undue reservation.
