# Supplementary material for: Effect of Tai Chi on the pain intensity or disability of patients with chronic low back pain: a systematic review and meta-analysis
Source: Front Sports Act Living. 2026 Feb 12;8:1676045. doi: 10.3389/fspor.2026.1676045 (PMC12935452; doi:10.3389/fspor.2026.1676045)
Supplement: Supplementary file 1 [file Datasheet1.docx]

Supplementary Material

**Supplementary Table 1.** Grading of Recommendations, Assessments, Developments and Evaluations (GRADE) approach for certainty in evidence

| **Certainty assessment** | | | | | | | **No of patients** | | **Effect** | | **Certainty** | **Importance** |
| --- | --- | --- | --- | --- | --- | --- | --- | --- | --- | --- | --- | --- |
| **No of studies** | **Study design** | **Risk of bias** | **Inconsistency** | **Indirectness** | **Imprecision** | **Other considerations** | **EG** | **CG** | **Relative (95% CI)** | **SMD (95% CI)** |  |  |
| VAS or NRS | | | | | | | | | | | | |
| 12 | randomized trials | not serious | serious | not serious | not serious | none | 502 | 402 | - | SMD −2.14 (−2.84 to −1.44) | ⨁⨁⨁◯ Moderate | CRITICAL |
| RMDQ | | | | | | | | | | | | |
| 14 | randomized trials | serious | serious | not serious | serious | none | 103 | 102 | - | SMD −1.45 (−2.49 to −0.40) | ⨁◯◯◯ Very Low | CRITICAL |
| ODI (Pain Intensity) | | | | | | | | | | | | |
| 3 | randomized trials | serious | serious | not serious | serious | none | 62 | 58 | - | SMD −1.99 (−2.94− to −1.04) | ⨁◯◯◯ Very Low | CRITICAL |
| ODI (Personal Care) | | | | | | | | | | | | |
| 3 | randomized trials | serious | not serious | not serious | serious | none | 62 | 58 | - | SMD −2.62 (−3.13 to −2.12) | ⨁⨁◯◯ Low | CRITICAL |
| ODI (Lifting) | | | | | | | | | | | | |
| 3 | randomized trials | serious | not serious | not serious | serious | none | 62 | 58 | - | SMD −1.91 (−2.35 to −1.47) | ⨁⨁◯◯ Low | CRITICAL |
| ODI (Walking) | | | | | | | | | | | | |
| 3 | randomized trials | serious | serious | not serious | serious | none | 62 | 58 | - | SMD −2.16 (−3.78 to −0.54) | ⨁◯◯◯ Very Low | CRITICAL |
| ODI (Standing) | | | | | | | | | | | | |
| 3 | randomized trials | serious | serious | not serious | serious | none | 62 | 58 | - | SMD −2.02 (−3.09 to −0.95) | ⨁◯◯◯ Very Low | CRITICAL |
| ODI (Sleeping) | | | | | | | | | | | | |
| 3 | randomized trials | serious | serious | not serious | serious | none | 62 | 58 | - | SMD −4.27 (−6.22 to −2.33) | ⨁◯◯◯ Very Low | CRITICAL |
| ODI (Sex Life) | | | | | | | | | | | | |
| 3 | randomized trials | serious | serious | not serious | serious | none | 62 | 58 | - | SMD −1.22 (−2.60 to 0.17) | ⨁◯◯◯ Very Low | CRITICAL |
| ODI (Sitting) | | | | | | | | | | | | |
| 3 | randomized trials | serious | serious | not serious | serious | none | 62 | 58 | - | SMD −2.59 (−4.11 to −1.07) | ⨁◯◯◯ Very Low | CRITICAL |
| ODI (Social Life) | | | | | | | | | | | | |
| 3 | randomized trials | serious | serious | not serious | serious | none | 62 | 58 | - | SMD −2.74 (−4.56 to −0.93) | ⨁◯◯◯ Very Low | CRITICAL |
| ODI (Travelling) | | | | | | | | | | | | |
| 3 | randomized trials | serious | serious | not serious | serious | none | 62 | 58 | - | SMD −2.57 (−3.96 to −1.18) | ⨁◯◯◯ Very Low | CRITICAL |

**Supplementary Table 2.** Leave-one-out sensitivity analysis for pain intensity

| **Omitted study** | **SMD (95% CI)** | **Heterogeneity Effect** | | **Overall Effect** | |
| --- | --- | --- | --- | --- | --- |
|  |  | **I^2^** | **P** | **Z** | **P** |
| Hall 2011 | -2.07 (-2.84, -1.29) | 93% | < 0.00001 | 5.21 | < 0.00001 |
| Li 2024 | -2.06 (-2.79, -1.33) | 94% | < 0.00001 | 5.51 | < 0.00001 |
| Liu 2019 | -2.10 (-2.84, -1.36) | 94% | < 0.00001 | 5.55 | < 0.00001 |
| Lu 2017 | -2.09 (-2.87, -1.31) | 94% | < 0.00001 | 5.27 | < 0.00001 |
| Muharram 2011 | -2.06 (-2.84, -1.29) | 93% | < 0.00001 | 5.22 | < 0.00001 |
| Tian 2013 | -2.10 (-2.84, -1.36) | 94% | < 0.00001 | 5.55 | < 0.00001 |
| Tong 2016 | -2.15 (-2.92, -1.38) | 94% | < 0.00001 | 5.47 | < 0.00001 |
| Tong 2017 | -2.38 (-2.83, -1.93) | 79% | < 0.00001 | 10.41 | < 0.00001 |
| Wang 2021 | -2.29 (-3.00, -1.57) | 93% | < 0.00001 | 6.30 | < 0.00001 |
| Wu 2013 | -2.03 (-2.77, -1.29) | 93% | < 0.00001 | 5.36 | < 0.00001 |
| Yan 2022 | -2.23 (-2.97, -1.50) | 93% | < 0.00001 | 5.97 | < 0.00001 |
| Zou 2019 | -2.10 (-2.84, -1.36) | 94% | < 0.00001 | 5.55 | < 0.00001 |

**Supplementary Figure 1.** Subgroup analysis of pain intensity by TC style


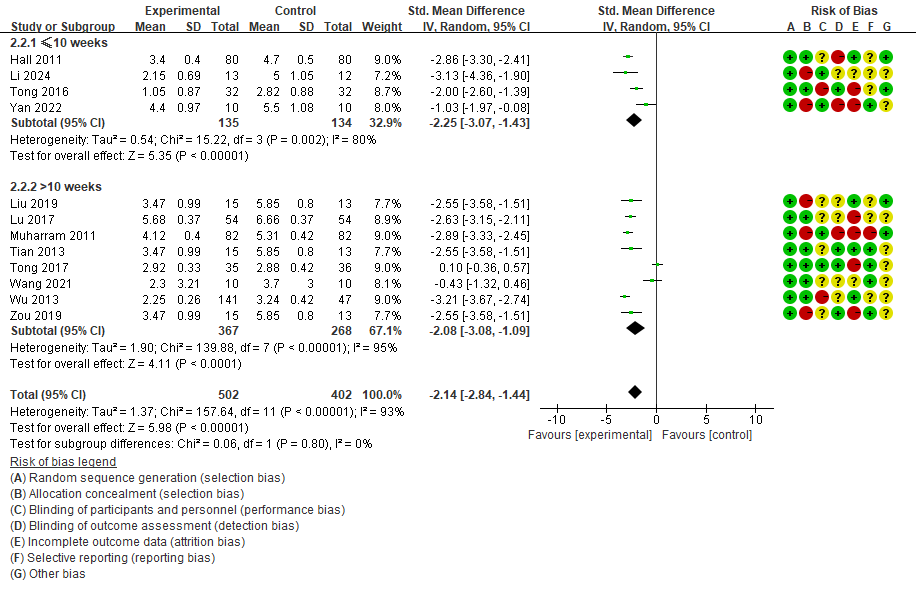


**Supplementary Figure 2.** Subgroup analysis of pain intensity by duration


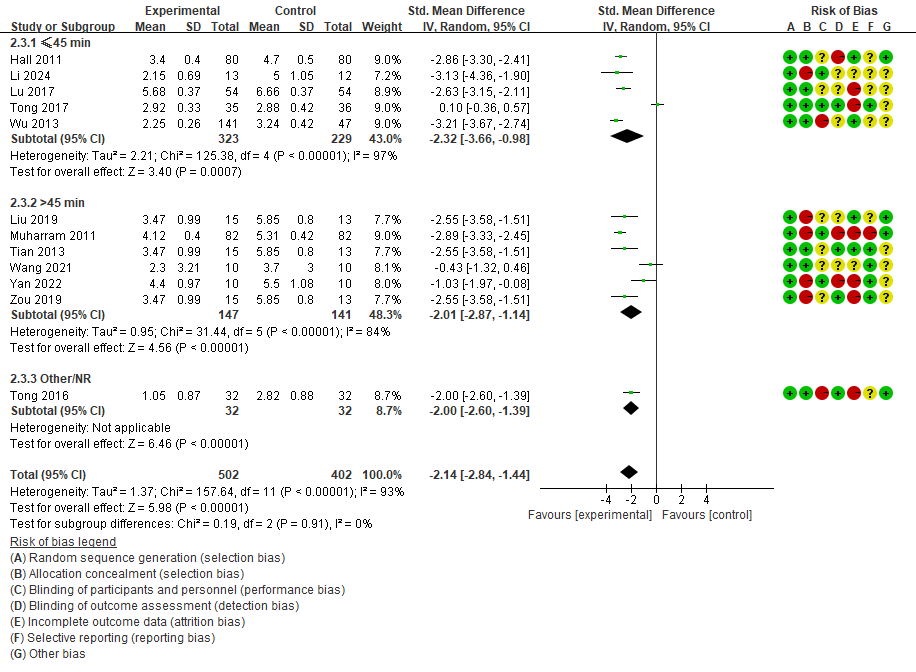


**Supplementary Figure 3.** Subgroup analysis of pain intensity by session


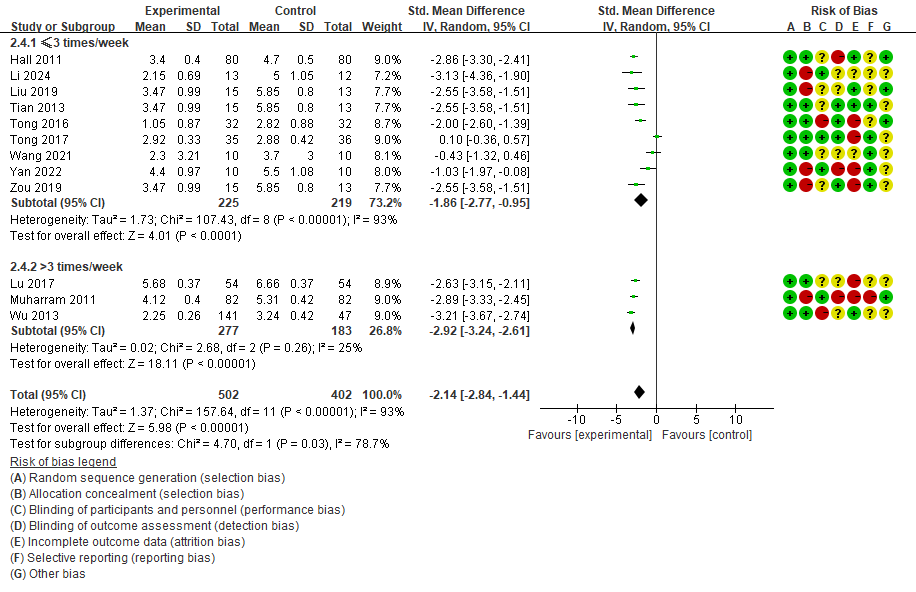


**Supplementary Figure 4.** Subgroup analysis of pain intensity by frequency


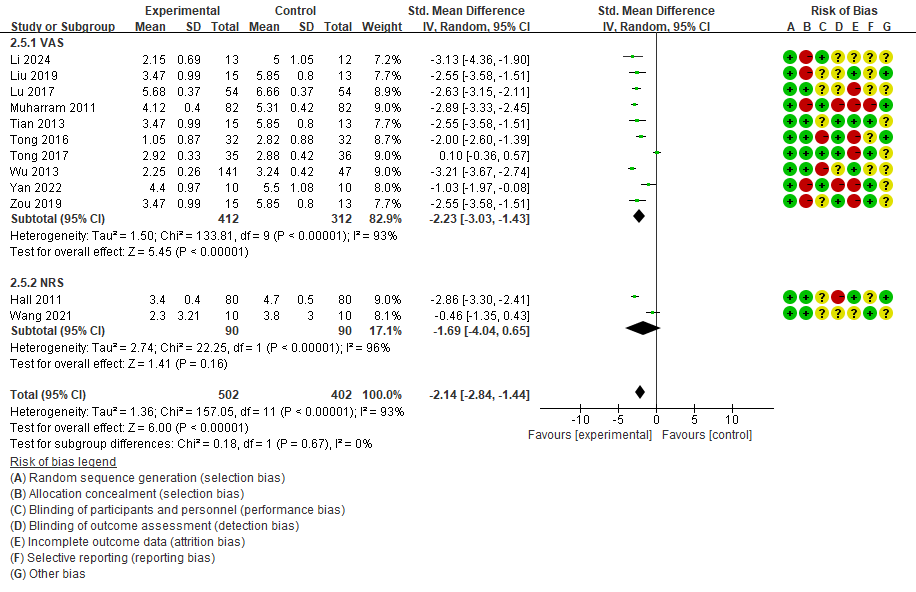


**Supplementary Figure 5.** Subgroup analysis of pain intensity by rating scale
